# Attentional variability and avoidance of hostile stimuli decrease aggression in Chinese male juvenile delinquents

**DOI:** 10.1186/s13034-021-00368-4

**Published:** 2021-04-13

**Authors:** Ziyi Zhao, Xianglian Yu, Zhihong Ren, Lin Zhang, Xu Li

**Affiliations:** 1 Key Laboratory of Adolescent Cyberpsychology and Behavior (CCNU), Ministry of Education, Key Laboratory of Human Development and Mental Health of Hubei Province, School of Psychology, Central China Normal University, No.152 Luoyu Road, Wuhan, Hubei 430079 People’s Republic of China; 2 Department of Education, Jianghan University, Wuhan, China

**Keywords:** Juvenile delinquents, Trial-level bias score, Hostile attention bias, Aggression, Antisocial

## Abstract

**Background:**

As a prominent issue worldwide, juveniles’ aggressive and violent crimes have attracted much interest in recent years. Based on the social information processing model, the present study aimed to evaluate the Chinese male juvenile delinquents’ attention bias towards hostile stimuli from both static and dynamic perspectives. Additionally, the predictive effect of attention bias on aggressive behavior and the moderating effect of group (juvenile delinquents and the controls with no criminal history) were also investigated.

**Methods:**

The hostile attention bias and aggressive behavior of 76 juvenile delinquents (*M*_age_ = 17.5 years, *SD* = 0.59 years) and 67 controls (*M*_age_ = 18.3 years, *SD* = 0.73 years) were measured with the emotional dot-probe task, emotional Stroop task, and the Chinese version of the Buss & Perry aggression questionnaire, respectively.

**Results:**

The results showed that compared with controls, juvenile delinquents showed more attention biases towards hostile faces and words, and demonstrated higher levels of physical aggression and anger. Furthermore, the type of participants moderated the relationship between hostile attention bias and aggressive behavior. For juvenile delinquents, attention bias away from hostile stimuli and attention variability negatively predicted anger, while for controls, attention variability positively predicted self-directed aggression.

**Conclusion:**

Attentional variability and avoidance of hostile stimuli are expected to reduce the aggressive level of Chinese male juvenile delinquents. The relationship between attention bias and aggression should be further considered and applied in the clinical practice.

## Background

Antisocial Personality Disorder (ASPD), characterized by failure to conform to social norms, a lack of remorse, impulsivity, irritability, and aggressiveness, mostly develops during childhood or early adolescence and continues into adulthood [1]. Juvenile delinquents who show aggressive and violent criminal behavior in adolescence are at higher risk of developing antisocial personality and may exhibit more serious criminal behavior in adulthood [2, 3]. Aggressive behavior is a frequent manifestation [4–6] and risk factor [7, 8] of antisocial personality disorder and lifelong criminals [9]. Furthermore, violent crime (including aggravated assault, rape, and robbery) is also the main form of juvenile delinquency in China and accounts for 73.38% of all types of juvenile crime in 2017 [10]. Violent crime among juvenile delinquents has received quite a bit of attention in the existing literature [8, 11, 12]. However, most of the existing studies are based on Western culture. Previous studies in the context of Eastern cultures have mostly focused on the aggressive behavior of the general adolescents or the adult criminals, but scarcely on juvenile delinquents [13].

Considering the severity of the status quo of juvenile violent crime in China, the importance of timely modification of aggression, and the scarcity of previous research, much attention should be paid to the Chinese juvenile delinquents and their maladaptive aggressive behaviors.

Among the impact factors of aggressive behavior, biased social cognition, especially the attention bias towards hostile stimuli, has been seen as a basis for the development and maintenance of aggression [14]. According to the social information processing model, selective attention allows us to focus on some information while filtering others and therefore has an important influence on the interpretation of social cues, emotion regulation [15, 16], and behaviors [14, 17]. Flexible and adaptive attention distribution can help individuals to pay more attention to positive cues, form positive interpretations of events, regulate negative emotions, and thus reduce aggressive responses [18]. Conversely, attention bias towards negative stimuli makes it easier for individuals to feel threatened, form hostile interpretations and generate hostile cognitive beliefs, which contributed to the development of general aggressive tendencies [19–21].

Many studies have explored the relationship between hostile attention bias and aggressive behavior, but the results were inconsistent [22]. On the one hand, studies on violent offenders reported a significant association between aggression and attention bias towards aggressive words [23–25], aggressive body language [26], angry faces [27] and threat stimuli [28, 29]. Similar findings have also been found in studies on aggressive adolescents [30], college students [31], and nonclinical male adults without psychological conditions [32]. On the other hand, studies on normal adults and aggressive children reported a significant association between attention bias away from hostile stimuli and aggression [18, 33, 34]. Studies on aggressive adolescents also showed that heightened reactive aggression scores were associated with suppressed rather than enhanced attention to hostile cues [35].

The inconsistency of these findings may result from the dynamic expression of attention and the reliability and sensitivity of the measures. Specifically, selective attention is not expressed in a stable or static manner but would change dynamically. As a multiple process expressed repeatedly and continuously over time, attention has different characteristics and functions in different phases [19] and shows multiple patterns across and within participants [36]. Instead of a stable or static process, attention bias has considerable temporal variability, which expresses in fluctuating, phasic bursts, toward or away from target stimuli over time [37]. The dynamic expression of attention processing has been supported by some studies among individuals with post-traumatic stress disorder [38, 39], anxiety disorder [40], and depression [41]. However, most of the previous studies measured the attention bias in a suboptimal way that may not reflect the dynamic nature of the attention process, which may lead to mixed findings. Considering the dynamic expression of attention, it is important to conceptualize and operationalize attention bias in a way that validly reflects the expression of attention processing.

The dot-probe task is one of the most common paradigms used to assess the attention bias, and the traditional bias score derived from the dot-probe task (Dot-BS) quantifies the attention bias by comparing reaction times of different trial types (consistent trials and inconsistent trials). However, the measurement of attention bias using Dot-BS showed poor split-half and test–retest reliabilities [42], and the dynamic expression of attention bias was considered as a possible explanation for the poor reliability of Dot-BS [37]. As an indicator derived from the traditional conceptualization of attention bias, Dot-BS can only reflect the attentional allocation at a certain time after the stimulus is presented and estimate the attention bias in a static and stable manner, which may not optimally reflect the time-series expression of attention bias. On the one hand, the static quantitative method is difficult to reflect dynamic attention characteristics comprehensively and truly. On the other hand, the averaging method might be easier to get null findings [39, 43]. Regarding the dynamic characteristic of attention bias, Zvielli and his colleges proposed Trial-level Bias Score (TLBS) to reflect the dynamic expression of attention bias [37]. By subtracting the reaction time of the consistent trial from that of the temporally contiguous inconsistent trial, TLBS can reflect the repeated and continuous allocation of the attention process to some extent. Some previous studies have reported that the TLBS can validly reflect the dynamic process of attention bias, and exhibits higher split-half reliability and predictive power than the Dot-BS [38, 39, 44]. Considering the important impacts of the conceptualization and quantification of attention bias, the present study would not only calculate the Dot-BS, but also utilize the TLBS to estimate the dynamic characteristics of attention allocation, and further explore the correlation between hostile attention bias and aggressive behavior.

The current study aimed to evaluate the characteristics of Chinese male juvenile delinquents’ attention bias towards hostile stimuli. Besides, the predictive effect of attention bias on aggressive behavior and the moderating effect of group (juvenile delinquents and the controls with no criminal history) were also investigated. Both hostile dot-probe task and emotional Stroop task were used to examine hostile attention bias. Additionally, in order to investigate the hostile attention bias of juvenile delinquents with antisocial tendency from both static and dynamic perspectives, we utilized not only the traditional quantification method but also the trial-level bias score. We hypothesized that greater attention bias towards hostile stimuli and a closer association between hostile attention bias and aggressive behavior would be found among juvenile delinquents rather than in controls.

## Methods

### Participants

Concerning the juvenile delinquents, the cluster sampling was adopted to recruit participants within a juvenile correctional institution in southeastern China. Three post-graduate students majoring in psychology conducted inclusion interviews with all juvenile delinquents who signed up for the study. The inclusion criteria were: (1) no history of mental disorders or attention disorders; (2) able to recognize words appropriately; (3) normal or corrected-to-normal vision and normal color vision. In order to control related confounding factors, no staff of juvenile correctional institution was involved in the recruitment and subsequent study. Regarding the recruitment of the control group, advertisements were posted at two universities in central China to recruit participants and the inclusion interviews and criteria were the same as those for juvenile delinquents.

Finally, eighty-four male juvenile delinquents (aged 16–18 years) were recruited from a juvenile correctional institution, with an average age of 17.5 years (*SD* = 0.59 years). Among them, 7.1% were sentenced to within one year, 73.2% between one to three years, and 19.7% between three to ten years. Sixty-nine non-criminal males (the control group) were recruited from two universities with an average age of 18.3 years (*SD* = 0.73 years).

This project was approved by the Ethics Committee of the Humanities and Social Sciences of Fuzhou University (EC2018021) and was registered (https://osf.io/e6fxv/). All participants participated in the study voluntarily and were told that they could withdraw from the study at any time for any reason. In addition, we assured the juvenile delinquents that the relevant research data and results would not be disclosed to the juvenile correctional institution. Informed consent was obtained from each participant, their legal guardians, and their guard unit (juvenile correctional institution) at the beginning of the study.

### Measures

#### Antisocial personality traits

The antisocial personality diagnostic subscale of the Chinese version of the Personality Diagnostic Questionnaire 4th edition-plus (PDQ-4 +) was used to measure the antisocial personality traits of juvenile delinquents [45]. It is a self-report questionnaire that consists of 8 true or false items (e.g., “Before the age of 15, did you intentionally abuse animals or hurt others?”), and the last item contains 15 sub-items. The participants received a score of 1 if more than 3 sub-items were chosen for the last item. According to Wang and his colleges, the criterion of all kinds of personality disorders adjusted to a score of 5–6 in Chinese adolescents [46]. In this study, the cut-off score of the antisocial personality disorder subscale of PDQ-4 + was 5. The PDQ-4 + showed satisfactory test reliability and validity in the Chinese sample. In the present study, the Cronbach’s α of PDQ-4 + was 0.86.

#### Aggressive behavior

The Chinese Version of the Buss & Perry Aggression Questionnaire (AQCV) was used to measure the participants’ overall level of aggression in areas of instrumental, affective, and cognitive, which exhibited in physical, verbal, anger, hostility, and self-directed aggression [47]. This scale consists of thirty items and includes five subscales: physical aggression (AQCV-PA) (e.g., “Once in a while, I can't control the urge to strike another person”), verbal aggression (AQCV-VA) (e.g., “I can’t help getting into arguments when people disagree with me”), anger (AQCV-A) (e.g., “I flare up quickly but get over it quickly”), hostility (AQCV-H) (e.g., “I am suspicious of overly friendly strangers”), and self-directed aggression (AQCV-SA) (e.g., “When I'm upset, I think about hurting myself”). Participants were instructed to rate each item on a 5-point Likert scale, from 1 (*completely untrue*) to 5 (*completely true*). The higher the score, the higher the level of aggression. In the present study, the Cronbach’s α of the subscales were as follows: Physical Aggression, 0.83; Verbal Aggression, 0.74; Anger, 0.70; Hostility, 0.72; Self-directed Aggression, 0.75; total score, 0.91.

#### Hostile attention bias

The emotional dot-probe task was employed to measure the attentional bias towards face expressions. Twenty pairs of hostile-neutral and twenty pairs of neutral–neutral face pictures were created by FaceGen3.4 (http://FaceGen.com; Singular Inversions, 2009) as the formal experimental materials. The experimental program included two parts: the practice phase (10 trials) and the formal experiment (120 trials). First, a gaze point (500 ms) was presented in the screen center, then a pair of face pictures (resolution 400 × 400) appeared on both sides of the gaze point (500 ms). A probe letter "E" appeared immediately on either side after the picture disappeared, then the participants were asked to indicate the location of the probe letter by clicking the mouse as quickly and accurately as possible. Attention bias towards hostile stimuli is characterized as responding to probes replacing threat facial expressions faster than responding to probes replacing neutral facial expressions [48]. Participants whose accuracy rate reached 80% or above in the practice session can start the formal experiment.

The emotional Stroop paradigm (eStroop) was utilized to assess the attention bias towards hostile and aggressive vocabularies. Forty hostile words were paired with forty neutral words. The experimental program contained the practice phase (10 trials) and the formal experiment (160 trials). At first, a gaze point (500 ms) was displayed on the screen, then the stimuli (the word printed in red or green color) was presented in the center of the screen. Participants were asked to ignore the meaning of the word and indicate the color of the word by pressing the keyboard as quickly and accurately as possible. Naming the colors of hostile-related words slower than naming the colors of neutral words indicates attention bias towards hostile stimuli [49]. Participants could begin the formal experiment only when their correct response rates were above 80%.

### Data preparation

The participants whose correct response rates were below 80% in the emotional dot-probe task or hostile eStroop task were excluded. In addition, we excluded the data of the practice stage, the incorrect response data of all participants, the data with the reaction time shorter than 200 ms or longer than 2000 ms in the correct response trials, and the data with the extreme reaction time exceeding the mean of all participants by three standard deviations. Finally, seventy-six juvenile delinquents and sixty-seven controls were included in further analysis. The demographic and crime information of juvenile delinquents is presented in Table 1.

**Table 1:** Crime information of juvenile delinquents

Variables	Juvenile delinquents (*n* = 76)
Type of Crime	
Property crime	38 (50.00%)
Violent crime	36 (47.40%)
Others	2 (2.60%)
Assigned time in the correctional institution	
Within one year	7 (9.20%)
One to three years (including three years)	45 (59.20%)
Three to ten years (including ten years)	24 (31.60%)

In the hostile eStroop task, the difference between the average reaction time of the hostile words and the average reaction time of the neutral words was calculated as attention bias towards hostile words (eStroop-BS). Scores of eStroop-BS above zero indicated attention bias toward hostile words, while scores below zero indicated attention bias away from hostile words.

In the emotional dot-probe task, the traditional bias score (Dot-BS) and trial-level bias score (TLBS) were measured.

Dot-BS: the average reaction time of incongruent trials (ITs, the position of the detection letter is different from that of the hostile face picture) minus the average reaction time of congruent trials (CTs, the position of the detection letter is the same as that of the hostile face picture).

TLBS: Referring to Zvielli, each CT was paired with a nearest IT which was temporally away (before or after) from the CT by no further than five trials, and the differences of reaction time between the contiguous pairs of CT and IT were calculated [37]. Five parameters can be further calculated based on the raw TLBSs: positive Mean TLBS (Mean-TLBS_toward_: mean of TLBSs > 0 ms, reflecting the participant’s mean attention bias towards target stimuli), negative Mean TLBS (Mean-TLBS_away_: mean of TLBSs < 0 ms, indicating the participant’s mean attention bias away from target stimuli), positive Peak TLBS (Peak-TLBS_toward_: maximum TLBS value per participant, reflecting the maximum value of participant's attention bias towards target stimuli), negative Peak TLBS (Peak-TLBS_away_: minimum TLBS value per person, indicating the maximum value of the participant's attention bias away from target stimuli), and variability in TLBS (TLBS-Variability: the sum of all distances between all sequential TLBSs divided by the total number of TLBSs, indicating the temporal variability of participant’s attention process with repeated alternations of attention bias towards or away from target stimuli).

It should be particularly noticed that negative TLBSs (Mean-TLBS_away_, Peak-TLBS_away_) are negative values representing the level of attention avoidance. Specifically, the larger its absolute value, the higher level of attention avoidance towards the hostile stimuli.

**Fig. 1:**
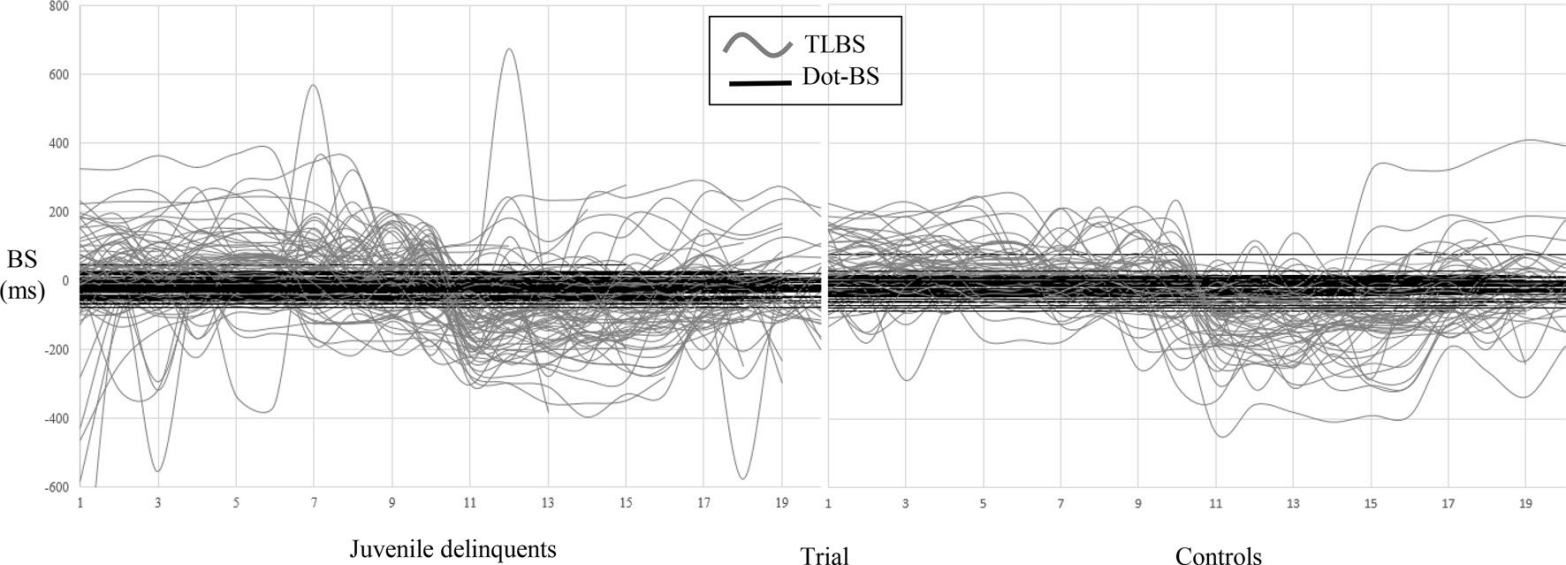
Comparison in Dot-BS and TLBS between juvenile delinquents and controls

The Dot-BS and TLBS of 76 juvenile delinquents and 67 controls are depicted in Fig. 1.

### Statistical analysis

(1) Pearson correlation analysis and independent-sample *t*-test were used to examine the correlation between demographic and primary outcomes, and estimate the differences between controls and juvenile delinquents; (2) Logistic regression was used to explore the predictive effect of hostile attention bias on group type (controls = 0; juvenile delinquents = 1); (3) Hierarchical regression analysis was then conducted to examine the predictive role of attention bias in aggressive behavior, as well as the moderating effect of group, and simple slope test was used to explore the specific interaction patterns.

## Results

### Preliminary analysis

Results of descriptive statistics and correlation analysis are displayed in Table 2. Independent-sample *t*-test revealed that the difference in age between juvenile delinquents and controls was significant, with juvenile delinquents younger than non-criminal controls. In terms of antisocial personality traits of juvenile delinquents, the scores of antisocial personality disorder subscale of PDQ-4 + ranged from 0 to 8 with a mean score of 3.25, which exceeds the cut-off score for the adult sample (score greater than 3). 29.8% of participants reached the cut-off score for the Chinese adolescent sample (score greater than 5).

**Table 2:** Mean, standard deviations, *t*-test, and correlation coefficients in the study

	1	2	3	4	5	6	7	8	9	10	11	12	13	14	15
1 Age	–	0.06	0.00	0.14	− 0.04	0.05	0.18	− 0.06	− 0.05	0.11	0.05	0.04	− 0.11	0.09	− 0.25^*^
2 AQCV	0.03	–	0.85^**^	0.79^**^	0.85^**^	0.77^**^	0.65^**^	0.05	0.16	− 0.23^*^	− 0.04	0.18	− 0.09	0.25^*^	− 0.23^*^
3 AQCV-PA	0.11	0.70^**^	–	0.61^**^	0.70^**^	0.46^**^	0.40^**^	0.08	− 0.23^*^	− 0.20	− 0.06	0.13	− 0.14	0.20	− 0.28^*^
4 AQCV-VA	− 0.09	0.76^**^	0.39^**^	–	0.62^**^	0.55^**^	0.45^**^	0.08	− 0.05	− 0.13	− 0.13	0.13	− 0.13	0.19	− 0.12
5 AQCV-A	− 0.03	0.73^**^	0.34^**^	0.54^**^	–	0.56^**^	0.44^**^	0.03	− 0.19	− 0.27^*^	− 0.01	0.24^*^	− 0.08	0.33^**^	− 0.30^**^
6 AQCV-H	0.08	0.82^**^	0.47^**^	0.54^**^	0.46^**^	–	0.49^**^	− 0.03	− 0.05	− 0.01	0.07	0.12	0.07	0.11	− 0.01
7 AQCV-SA	− 0.04	0.67^**^	0.26^*^	0.48^**^	0.47^**^	0.44^**^	–	0.03	− 0.05	− 0.29^**^	− 0.07	0.07	− 0.09	0.13	− 0.16
8 Dot-BS	0.14	0.04	0.09	− 0.09	− 0.02	0.06	0.05	–	− 0.02	− 0.09	0.16	0.50^**^	0.16	0.47^**^	− 0.21
9 eStroop-BS	0.18	0.07	0.06	0.02	− 0.15	0.18	0.09	− 0.02	–	0.10	0.21	− 0.20	0.19	− 0.17	0.31^**^
10 N-RT	0.18	0.18	0.23	0.10	− 0.07	0.22	0.13	0.00	0.18	–	0.17	− 0.25^*^	0.22	− 0.35^**^	0.43^**^
11 Mean-TLBS_toward_	0.30^*^	0.06	0.06	0.13	0.03	− 0.01	0.03	0.23	0.14	0.26^*^	–	0.00	0.80^**^	0.03	0.30^**^
12 Mean-TLBS_away_	− 0.18	− 0.05	0.02	− 0.10	0.05	− 0.09	− 0.08	0.14	− 0.35^**^	− 0.48^**^	− 0.25^*^	–	− 0.07	0.86^**^	− 0.62^**^
13 Peak-TLBS_toward_	0.32^**^	0.04	0.08	0.16	− 0.02	− 0.02	− 0.02	0.23	0.09	0.28^*^	0.91^**^	− 0.21	–	− 0.12	0.57^**^
14 Peak-TLBS_away_	− 0.15	− 0.07	0.00	− 0.06	0.03	− 0.10	− 0.12	0.14	− 0.30^*^	0.54^**^	− 0.20	0.93^**^	− 0.12	–	− 0.73^**^
15 TLBS-V	0.13	0.16	0.03	0.18	0.08	0.13	0.20	0.01	0.30^*^	0.60^**^	0.48^**^	− .80^**^	.41^**^	− 0.84^**^	–
*M(SD)*-J	17.54 (0.55)	73.33 (19.21)	19.77 (7.11)	12.49 (3.50)	14.04 (4.85)	15.83 (5.08)	11.21 (3.57)	− 19.49 (31.86)	3.03 (22.08)	389.58 (60.90)	75.06 (51.88)	− 95.12 (64.12)	156.17 (116.06)	− 202.36 (148.49)	55.10 (29.24)
*M(SD)*-C	18.25 (0.73)	69.24 (15.43)	15.64 (4.87)	11.70 (3.13)	12.66 (3.76)	17.97 (5.47)	11.27 (3.50)	− 21.15 (25.98)	7.77 (16.06)	358.24 (63.04)	57.70 (39.96)	− 86.12 (49.59)	112.27 (78.12)	− 172.61 (105.41)	45.37 (22.06)
*t*	6.76^***^	1.40	4.12^***^	1.42	1.93	− 2.44^*^	− 0.11	0.34	-1.45	3.04^**^	2.27^*^	− 0.93	2.63^**^	− 1.19	2.23^*^
Cohen’s *d*	1.10	0.23	0.68	0.24	0.32	0.41	0.02	0.06	0.25	0.51	0.37	0.16	0.44	0.23	0.38

***p < 0.001; **p < 0.01; *p < 0.05. In the correlation matrix, below the diagonal are the correlation coefficients among the controls; above the diagonal are the correlation coefficients among the juvenile delinquents. *AQCV* Chinese version of Buss & Perry aggression questionnaire, *AQCV-PA* AQCV-Physical Aggression, *AQCV-VA* AQCV-Verbal Aggression, *AQCV-A* AQCV-Anger, *AQCV-H* AQCV- Hostility, *AQCV-SA* AQCV-Self-directed Aggression, *Dot-BS* traditional bias score derived from the dot-probe task, *eStroop-BS* bias score derived from emotional Stroop, *N-RT* the reaction time of neutral trial, *Mean-TLBS*_*toward*_ mean attention bias score towards hostile stimuli, *Mean-TLBS*_*away*_ mean attention bias score away from hostile stimuli, *Peak-TLBS*_*toward*_ peak attention bias score towards hostile stimuli, *Peak-TLBS*_*away*_ peak attention bias score away from hostile stimuli, *TLBS-V* the temporal variability of attention process, *M(SD)-J* Mean and standard deviation of juvenile delinquents, *M(SD)-C* Mean and standard deviation of controls

Regarding attention bias, the positive TLBSs (Mean-TLBS_toward_, Peak-TLBS_toward_), TLBS-Variability, and reaction time of juvenile delinquents in neutral trials (N-RT) were significantly higher than those of controls. In terms of aggression, the AQCV-PA scores of juvenile delinquents were significantly higher than that of controls, but the AQCV-H scores of delinquents were significantly lower than that of controls. Considering the significant differences in age and N-RT between juvenile delinquents and controls, these variables were entered as covariates in further analysis.

### The predictive role of hostile attention bias in the group

The logistic regression was conducted to examine the predictive effect of hostile attention bias on group. Age and N-RT were entered as covariates, indicators of attention bias (i.e., the Dot-BS, TLBSs, eStroop-BS respectively) as the independent variables, and the group (control = 0, juvenile delinquents = 1) as the dependent variable. Results revealed that the predictive effect of Dot-BS or eStroop-BS on the group was not significant. The Mean-TLBS_toward_ and Peak-TLBS_toward_ can marginally predict the group (*OR* = 1.01, *p* = 0.04, 95% CI = 1.00–1.02; *OR* = 1.01, *p* = 0.05, 95% CI = 1.00–1.01).

### Aggressive behavior predicted by hostile attention bias: the moderating effect of group

The association between hostile attention bias and aggressive behavior as well as the moderating role of the group were investigated using multiple regression analysis. The independent variables and the control variables were first centralized and the groups were dummy encoded (control = 0, juvenile delinquents = 1). Age and N-RT were entered as control variables. Attention bias indicators (Dot-BS, TLBSs, eStroop-BS), group, and their interactions were included as predictor variables. Six models were constructed separately, with AQCV total scores and the five sub-scores as the dependent variable, respectively. The results of collinearity diagnostics found that the tolerance of each model was above 0.26, and the variance inflation factor (VIF) was below 3.88, suggesting no evidence of multicollinearity.

The results of multiple regression are shown in Table 3. After controlling the effect of age and N-RT, the interaction of group and TLBS-Variability can predict the total score of AQCV (β = 0.12, *t* = − 2.10, *p* = 0.037, 95% CI = − 0.50 to − 0.02); AQCV-SA score (β = − 0.34,* t* = − 2.22, *p* = 0.028, 95% CI = − 0.10 to − 0.01); and AQCV-A (β = − 0.37,* t* = − 2.52, *p* = 0.013, 95% CI = − 0.13 to − 0.02), respectively. Peak-TLBS_away_ and its interaction with group were marginally predictive of AQCV-A score (β = 0.40,* t* = 1.95, *p* = 0.054, 95% CI = 0.000–0.023).

**Table 3:** Hierarchical regression analysis for aggression and attention bias

Independent variable	AQCV				AQCV-SA				AQCV-A			
	*B*	β	*t*	*△R*^*2*^	*B*	β	*t*	*△R*^*2*^	*B*	β	*t*	*△R*^*2*^
Step 1				0.00				0.01				0.03
Age	1.10	0.05	0.45		0.29	0.06	0.60		0.02	0.00	0.03	
N-RT	− 0.01	− 0.05	− 0.51		− 0.01	− 0.16	− 1.60		− 0.01	− 0.20	− 2.02	
Step 2				0.02				0.01				0.04*
Group	5.15	0.15	1.46		0.29	0.04	0.40		1.72	0.20	2.04*	
TLBS-V	0.13	0.20	1.22		0.05	0.35	2.10*		0.04	0.23	1.46	
Step 3				0.03*				0.03*				0.04*
Group × TLBS-V	− 0.26	0.12	− 2.10*		− 0.05	− 0.34	− 2.22*		− 0.07	− 0.37	− 2.52*	

**p < 0 .01; *p < 0.05. Group were dummy encoded (controls = 0; juvenile delinquents = 1); *AQCV* Chinese version of Buss & Perry aggression questionnaire, *AQCV-SA* AQCV Self-directed Aggression, *AQCV-A* AQCV-Anger, *N-RT* the reaction time of neutral trial, *TLBS-V* the temporal variability of attention process, *Group × TLBS-V* the interaction of group and TLBS-Variability

**Fig. 2:**
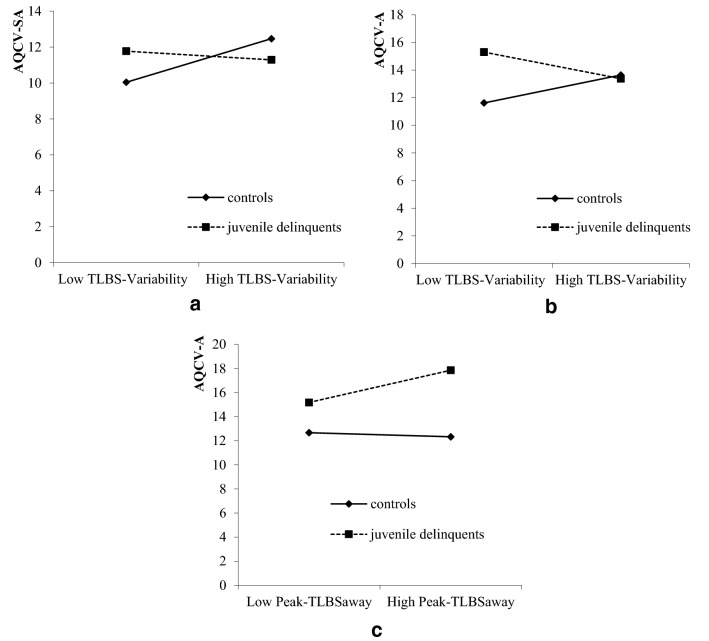
Interaction effect of TLBS scores and group on AQCV sub-scores

Further simple slope tests were conducted to examine the specific interaction patterns between the group and hostile attention bias (Fig. 2). For AQCV-SA scores, TLBS-Variability scores of controls were positively predictive of their AQCV-SA scores (*simple slope* = 0.05, *t* = 2.10, *p* = 0.04), but the predictive effect of TLBS-Variability was not significant for juvenile delinquents (*simple slope* = 0.01, *t* = − 0.42, *p* = 0.68) (Fig. 2a). For AQCV-A scores, TLBS-Variability scores were marginally negatively predictive of AQCV-A scores of juvenile delinquents (*simple slope* = − 0.04, *t* = − 2.02, *p* = 0.05), but the predictive effect was not significant among controls (*simple slope* = 0.04,* t* = 1.46, *p* = 0.15) (Fig. 2b). Besides, the interaction of group and Peak-TLBS_away_ scores was significantly predictive of AQCV-A scores, which showed that for controls the association between Peak-TLBS_away_ and AQCV-A was not significant (*simple slope* = − 0.00,* t* = − 0.25, *p* = 0.81), while for juvenile delinquents Peak-TLBS_away_ scores were positively predictive of AQCV-A scores (*simple slope* = 0.01,* t* = 2.84, *p* = 0.003) (Fig. 2c). That was to say, for juvenile delinquents, the higher level of attentional avoidance of hostile stimuli, the lower level of anger.

## Discussion

As a worldwide issue, violent crime among juvenile delinquents has received quite a bit of attention [8, 11, 12]. The current study examined Chinese male juvenile delinquents’ attention bias towards hostile stimuli, the predictive role of attention bias in aggressive behavior, and the moderating effect of group (juvenile delinquents and controls). We found that male juvenile delinquents with antisocial tendency reported more physical aggression and higher levels of anger and attention bias towards hostile faces compared to male controls. Positive TLBSs (Mean-TLBS_toward_, Peak-TLBS_toward_) can marginally predict the group type of participants. Individuals who had higher levels of attention bias towards hostile stimuli were prone to become juvenile delinquents. Besides, group could moderate the association between attention bias and aggressive behavior. For juvenile delinquents, attentional variability and avoidance of hostile stimuli negatively predicted AQCV-A scores. For controls, attentional variability positively predicted AQCV-SA scores.

### Juvenile delinquents’ attention bias and hostile characteristics

The Dot-BS showed that juvenile delinquents with antisocial tendency exhibited attentional avoidance of hostile stimuli, however, TLBS revealed that juvenile delinquents’ attention bias towards hostile stimuli varied temporally, fluctuating from attention bias towards stimuli to away from stimuli over time, and the feature of attention bias towards hostile stimuli could help distinguish juvenile delinquents from controls. The subsequent analysis showed that TLBSs, rather than Dot-BS, were effective predictors of the association between hostile attention bias and aggressive behavior. The result suggests that, although juvenile delinquents exhibit attention bias away from hostile stimuli statically and stably, the hostile attention bias they exhibit during the dynamic expression of attention may be their key cognitive feature. Compared with the static conceptualization and quantification of attention bias, the dynamic perspective may reveal the characteristic and processing of attention bias in a more integrated and comprehensive way [37].

Inconsistent with our hypothesis, the present study showed that the hostility scores of delinquents were significantly lower than those of controls. The measurement scale for hostility used in the current study may be the main reason for this inconsistency. In the present study, AQCV- Hostility subscale was used to measure participants’ hostility, which consists of seven descriptive statements beginning with “I” (e.g., I am sometimes eaten up with jealousy). Participants are told to indicate to what extent each statement can describe them [47]. Compared with controls, juvenile delinquents have less awareness and understanding of themselves, are more likely to believe that others need to be changed [50], and attribute problems to themselves less frequently. Thus, juvenile delinquents may score lower on those descriptions when self-reporting. Controls have a more objective and clear understanding of themselves, and a clearer awareness of their own characteristics and attribution patterns. Therefore, controls' scores on those descriptions may be more in line with their real levels. In other words, the self-reported AQCV-A subscale might underestimate juvenile delinquents’ hostility level.

### The predictive effect of juvenile delinquents’ hostile attention bias on aggressive behavior

The moderation effect analysis revealed that Peak-TLBS_away_ score can positively predict AQCV-A among juvenile delinquents. Peak-TLBS_away_ (the minimum TLBS value per person) is a negative value, and the smaller its score, the larger its absolute value, the higher level of attention avoidance towards the hostile stimuli. Therefore, the positive prediction effect of Peak-TLBS_away_ on AQCV-A means that the smaller Peak-TLBS_away_ (the higher level of attention avoidance), the smaller of AQCV-A scores (the lower level of anger). Hostile attention bias is regarded as an automatic process, but the sensory system can select stimuli by top-down attentional control, which can help regulate emotion [16]. In line with this view, the emotion regulation model holds that attention bias away from hostile stimuli can help decrease the arousal level of the sympathetic nervous system and regulate negative emotions, and further improve the adaptability of individuals in the threatening situation [51, 52]. Previous studies have also proved that the strategy of distraction is helpful to decrease reactive aggression [53] and anger [54], and aggressive individuals could reduce interpersonal conflicts by avoiding eye-contacting [33]. Therefore, attentional avoidance of threat stimuli could be considered as an effective emotion regulation strategy to relieve negative emotions, which in turn can reduce anger.

Furthermore, the negative prediction of AQCV-A scores by juvenile delinquents’ TLBS-Variability scores may further reflect long-term attention allocation disposition of emotion regulation in juvenile delinquents. Previous studies on spider phobia revealed that individuals may regulate the negative emotion by dynamically changing the attention direction [37]. They manifested firstly hypervigilance to hostile stimuli, followed by strategic avoidance to decrease fear and anxiety. This process repeated over time and finally showed a high level of attention variability. Similarly, we found that juvenile delinquents with antisocial tendency manifested a dynamic alternation of attention bias toward and away from hostile stimuli, and the variability of attention may reflect the anger regulation by controlling attention allocation. Therefore, juvenile delinquents who have a high level of attention variability can decrease anger and aggression by using more efficient emotion regulation strategies.

### The influence of culture and social context

As mentioned above, the present study reported a negative association between attention avoidance/variability and anger. This is different from the finding of studies based on Western samples, which reported a positive association between hostile attention bias and aggression [30, 23, 24, 26, 29]. In this regard, we believe that the Eastern culture and specific social context (juvenile correctional institution), characterized by collectivism, may suppress juvenile delinquents’ attention bias towards hostile stimuli, and induce their attention avoidance and variability for the adaptation of the specific environment in the juvenile correctional institution. On the one hand, Eastern culture emphasizes the importance of harmonious relationships, social roles, a sense of belonging and authority. Individual’s social information processes and behaviors are significantly influenced, restricted, and shaped by the social norms and values, which provide a framework to decide which behaviors and emotions are acceptable and valued, and which are unacceptable and undesirable [55]. Combined with the characteristics of Eastern culture, the special context of the juvenile correctional institution characterized as strict institutional norms, a clear hierarchy of power, and clear rewards and punishments, can shape individuals’ cognition and behaviors directly. Juvenile delinquents are divided into different groups. If the behavior of a single individual affects the honor of the whole group, the whole group will be punished or rewarded. Such settings can amplify the effects of norms, which in turn can constrain individual behavior. In addition, the specific social norms can also shape the popular peer norms, then further affect an individual’s social status [56]. People who conform to institutional norms are more likely to be rewarded and thus acquire a higher level of social status, and vice versa. In the juvenile correctional institution, the hostile attention bias and the corresponding aggressive behavior are maladaptive and thus suppressed, while relevant educational programs in the institution, such as social skills and interpersonal communication training, could be used to guide the discipline of juvenile delinquents and could help develop adaptive information processing and behaviors. Overall, the characteristics of Eastern culture and the rules in the juvenile correctional institution may suppress juvenile delinquents’ attention bias towards hostile stimuli, and instead, prompt the attention avoidance and variability, and finally reduce the level of anger. Considering the shaping effect of cultural and social contexts on cognition and behavior, it is necessary to conduct further cross-cultural research in the future.

### Limitations and implications

The current study has some limitations. Firstly, the sample size was relatively small, and there might be sampling bias. It is necessary to increase the sample size in future studies. Secondly, some of the findings were reported with p-values equal to 0.05, not less than 0.05, therefore, it is necessary to conduct replication studies to verify the current findings. Thirdly, the present study indicated that the attentional variability of controls, different from that of the juvenile delinquents, was positively predictive of self-directed aggression. One possible reason for this difference may be that the cognitive processes and functions reflected by attention variability are discrepant in different groups [57]. However, the present study failed to further verify this argument. It is necessary for future studies to further explore the cognitive processes and functions of attention indicators in different participant groups, which is of great significance both theoretically and clinically. Forthly, as the first step of social information processing, the encoding procedure can further be divided into autonomic processing and strategic processing [58], and their relationships with aggressive behavior are different between the early and late stages of processing [19]. The present study only focused on the late stage of information encoding but ignored the early stage. Therefore, in future research, it is necessary to take the attention processing phase into account and adopt a subconscious autonomic processing method simultaneously. In this way, we can further investigate attention encoding processing and its association with aggressive behavior in a more integrated and comprehensive way. Finally, the current study only included male juvenile delinquents. It is worth noting that there are significant differences between male and female juvenile delinquents in the level of delinquent behavior, crime seriousness, emotional symptoms, and psychopathic traits [59]. Whether the current results can be extrapolated to the sample of female juvenile delinquents need to be validated.

Notwithstanding these limitations, the results of the present study have great implications for clinical practice. We can intervene juvenile delinquents’ aggressive behaviors by targeting attentional avoidance of hostile stimuli, from the “down-top” or “top-down” perspective. On one side, from the “down-top” perspective, we could target implicit and automatic attention processing to reinforce attention towards non-hostile stimuli, and further adjust the behavior. Attention Bias Modification (ABM) is an intervention program that targets specific attention bias patterns [60]. By training the individuals to pay more attention to the positive target stimuli and suppress the attention bias towards hostile stimuli, ABM helps people to improve maladaptive emotional and behavioral responses [61, 62]. Furthermore, ABM can also be well combined with the internet, to meet the structured, low-cost, and high-efficiency intervention requirements for aggressive adolescents. On the other side, researchers could intervene the attention processing from a “top-down” perspective, namely decreasing attention bias towards threat bias through improving individuals’ cognitive control. As the most widely used treatment for aggression [63], Cognitive-Behavioral Intervention (CBI) has been reported with a potential application in reducing aggression level in adolescents [64, 65]. Specifically, CBI could be applied in juvenile correctional institutions to reduce aggressive levels in high-risk adolescents through top-down attentional control.

## Conclusion

Compared with controls, juvenile delinquents showed more attention biases towards hostile stimuli and demonstrated higher levels of physical aggression and anger. In addition, the type of participants moderated the relationship between hostile attention bias and aggressive behavior. For juvenile delinquents, attention bias away from hostile stimuli and attention variability negatively predicted anger, while for controls, attention variability positively predicted self-directed aggression.

## Data Availability

The datasets used and/or analyzed during the current study are available from the corresponding author on reasonable request.

## Abbreviations

Dot-BS: The traditional bias score derived from the dot-probe task
TLBS: Trial-level bias score derived from the dot-probe task
eStroop-BS: Bias score derived from eStroop-BS
IT: The position of the detection letter was different from that of the hostile face picture
CT: The position of the detection letter was the same with that of the hostile face picture
Mean-TLBS_toward_: Mean attention bias score towards hostile stimuli
Mean-TLBS_away_: Mean attention bias score away from hostile stimuli
Peak-TLBS_toward_: Peak attention bias score towards hostile stimuli
Peak-TLBS_away_: Peak attention bias score away from hostile stimuli
TLBS-Variability: The temporal variability of attention process
N-RT: Reaction time in neutral trials
AQCV: Chinese version of Buss & Perry aggression questionnaire
AQCV-PA: AQCV-Physical Aggression
AQCV-VA: AQCV-Verbal Aggression
AQCV-A: AQCV-Anger
AQCV-H: AQCV- Hostility
AQCV-SA: AQCV Self-directed Aggression
